# Flagellar Hook Protein FlgE Induces Microvascular Hyperpermeability *via* Ectopic ATP Synthase β on Endothelial Surface

**DOI:** 10.3389/fcimb.2021.724912

**Published:** 2021-11-02

**Authors:** Yuanyuan Li, Ying Shen, Yudan Zheng, Shundong Ji, Mengru Wang, Beibei Wang, Qingzhen Han, Yufeng Tian, Yiqiang Wang

**Affiliations:** ^1^The MOH Key Lab of Thrombosis and Hemostasis, The First Affiliated Hospital of Soochow University, Soochow University, Suzhou, China; ^2^Department of Laboratory Examination, People’s Hospital of Rizhao City, Rizhao, China; ^3^Center for Informational Biology, School of Life Science and Technology, University of Electronic Science and Technology of China, Chengdu, China; ^4^Department of Laboratory Examination, The First Affiliated Hospital of Soochow University, Soochow University, Suzhou, China; ^5^Central Lab, Xiang’an Hospital of Xiamen University, Xiamen University Medical Center, Xiamen University, Xiamen, China

**Keywords:** ATP synthase subunit β, ATP5B, flagellar hook protein, FlgE, vascular endothelial cells

## Abstract

We previously demonstrated the immunostimulatory efficacy of *Pseudomonas aeruginosa* flagellar hook protein FlgE on epithelial cells, presumably *via* ectopic ATP synthases or subunits ATP5B on cell membranes. Here, by using recombinant wild-type FlgE, mutant FlgE (FlgEM; bearing mutations on two postulated critical epitopes B and F), and a FlgE analog in pull-down assay, Western blotting, flow cytometry, and ELISA, actual bindings of FlgE proteins or epitope B/F peptides with ATP5B were all confirmed. Upon treatment with FlgE proteins, human umbilical vein endothelial cells (HUVECs) and SV40-immortalized murine vascular endothelial cells manifested decreased proliferation, migration, tube formation, and surface ATP production and increased apoptosis. FlgE proteins increased the permeability of HUVEC monolayers to soluble large molecules like dextran as well as to neutrophils. Immunofluorescence showed that FlgE induced clustering and conjugation of F-actin in HUVECs. In Balb/c-nude mice bearing transplanted solid tumors, FlgE proteins induced a microvascular hyperpermeability in pinna, lungs, tumor mass, and abdominal cavity. All effects observed in FlgE proteins were partially or completely impaired in FlgEM proteins or blocked by pretreatment with anti-ATP5B antibodies. Upon coculture of bacteria with HUVECs, FlgE was detectable in the membrane and cytosol of HUVECs. It was concluded that FlgE posed a pathogenic ligand of ectopic ATP5B that, upon FlgE–ATP5B coupling on endothelial cells, modulated properties and increased permeability of endothelial layers both *in vitro* and *in vivo*. The FlgE-ectopic ATP5B duo might contribute to the pathogenesis of disorders associated with bacterial infection or ectopic ATP5B-positive cells.

## Introduction

*Pseudomonas aeruginosa* (PA) is a common pathogen in sites like the lungs or eyes or exposed cutaneous injuries but can also be transmitted *via* blood circulation to other organs (Bachta et al., 2020). The interactions between PA and host cells/molecules remain to be fully characterized. In a project addressing host–pathogen interactions, we found that PA flagellar hook and its composition of monomers, namely, flagellar hook FlgE proteins (NP_249771), represented a new type of pathogen-derived molecules that stimulated a proinflammatory response in cultured cells or in living mice (Shen et al., 2017; Li et al., 2019). Structural analysis and experiments suggested that epitopes B (aa168-174, PPTVTPF) and F (aa303-309, TPPTYAW) on FlgE surface were required for FlgE bioactivity (Li et al., 2019). In an effort to identify receptors or molecules that mediated FlgE immunostimulation, total membrane proteins were prepared from epithelial cells and subjected to pull-down assay against recombinant FlgE proteins. Mass spectrometry of FlgE-binding proteins revealed that ATP synthase subunits β (ATP5B), α (ATP5A), O (ATP5O), and caveolin-1 were the top four promising candidates for binding FlgE proteins (Shen et al., 2017). Since ATP synthase or its subunit ATP5B had been previously found on the surface of several types of cells like hepatocytes, cancer cells, or vascular endothelial cells (VECs) as receptors of several ligands (Das et al., 1994; Chi and Pizzo, 2006; Taurino and Gnoni, 2018), the abovementioned pull-down/mass spectrometry results prompted us to suspect that PA FlgE also utilized these ectopic (ecto) ATP synthase or their subunits to modulate properties of cells that express ectopic ATP synthase cells. Should the proposed FlgE–ATP synthase/subunit interactions actually occur in living organisms like in humans, it would advance our understanding of bacteria–host interactions in a few conditions concerning ectopic ATP synthase-expressing cells, such as in vascular endothelium. Though it still remains unclear why, how, in what cells, or under what situation would ATP synthase relocate from its traditional destination (e.g., inner membrane of mitochondria) to the outer surface of cellular membrane, ectoATP5B on VECs had been reported to mediate, at least partially, cellular responses to several host-derived molecules like angiostatin (Moser et al., 1999), apolipoprotein (Apo) A-I (Radojkovic et al., 2009; Cavelier et al., 2012), kringle 1-5 of plasminogen (Veitonmaki et al., 2004), or pigment epithelium-derived factor (Notari et al., 2010). To the best of our knowledge, the only pathogen-associated molecule with a clear identity that bound ectoATP5B was bacterial adhesin FimH (Shin and Kim, 2010). Besides, ectoATP5B had been proposed to participate in a couple of other pathogen-associated processes. Yavlovich et al. noted that during transfer of HIV-1 from dendritic cells to CD4^+^ cells, ectoATP5B proteins were clustered in the immune synapse (Yavlovich et al., 2012), while Ahmed et al. showed that hepatitis E virus utilized ectoATP5B to enter hepatocytes (Ahmed et al., 2016). Liang et al. found that an ATP5B-homologue protein expressed on the surface of shrimp gill cell membrane was able to bind white spot syndrome virus particles (Liang et al., 2010). In the last three cases, the exact molecular identities of the viral constituents that bound ectoATP5B remained to be identified. While better understanding of pathogen–host interactions has always been a focus of microbiology and immunology, we tried to clarify whether FlgE would act as an alternative ligand of ectoATP5B on VECs. On the other hand, quite a few critical disorders base their pathogenesis heavily on VEC biology, such as atherosclerosis, sepsis, and cytokine storm. Thus, investigation into FlgE effect on VECs would advance our understanding of those disorders concerning both infections and vascular biology. To achieve this goal, the effect of FlgE on systemic vascular permeability was also studied by using an animal model.

## Materials and Methods

### Cell Culture

Human umbilical vein endothelial cell (HUVEC), SV40-transformed endothelial cells of mouse (SVECs), and human hepatocellular cancer (HepG2) cell lines were from China Center for Type Culture Collection. HUVECs were cultured in Roswell Park Memorial Institute (RPMI)-1640 medium, while SVECs and HepG2 cells were cultured in Dulbecco’s modified Eagle’s medium (DMEM). If not specified, both media were supplemented with 10% (v/v) heat-inactivated fetal bovine serum (FBS), 100 μg/ml of penicillin, and 100 μg/ml of streptomycin; and cultures were performed at 37°C in 5% CO_2_/95% air.

### Recombinant FlgE or Mutant FlgE Proteins and Synthetic Peptides

Recombinant PA wild-type FlgE proteins or FlgEM proteins (with mutations on epitopes B and F), both with 6× His Tag at C-terminals, were obtained and proven to be free from endotoxin contaminant as described before (Lin et al., 2013; Shen et al., 2017). In brief, to generate FlgEM proteins, site B of FlgE (NP_249771, aa168-174, PPTVTPF) was deleted and site F (NP_249771, aa303-309, TPPTYAW) was mutated to “AAA” *via* routine engineering. Peptides Pc-B and Pc-F corresponding to wild-type epitopes B and F, respectively, were synthesized with or without fluorescein isothiocyanate (FITC) fluorescein labeling at N-terminal (Shanghai Biotech BioScience and Technology Company, Shanghai, China).

### Generation and Purification of Polyclonal Rabbit Anti-ATP5B or Anti-FlgE Antibodies

To generate anti-ATP5B antibodies for blocking assays, an ATP5B analog protein (i.e., a recombinant protein containing a tandem of predicted B epitopes, or “rATP5BesT” in short) was designed, expressed in *Escherichia coli*, purified, and then used for immunizing New Zealand White rabbits; and anti-ATP5B antibodies were purified, all as described earlier (Wang et al., 2019). In essence, this antibody batch was able to bind or label surface of recombinant or *in situ* ATP5B *de novo*. Anti-FlgE antibodies were prepared with a similar protocol using recombinant FlgE as immunogens (**Supplementary Material/Methods**).

### Confirmative Pull-Down Assay of Hypothetical FlgE–ATP5B Interaction

Purified recombinant His-Tagged FlgE or FlgEM proteins measuring at 500 μg were incubated with 30 μl of Ni-NTA beads (GE Healthcare, Uppsala, Sweden) for 8 h at 4°C on a rocker; and unbound FlgE or FlgEM was washed away with binding buffer of 30 mM of imidazole (Sigma, St Louis, MO). Subsequently, the FlgE-saturated beads were incubated for 8 h at 4°C with 200 μg of plasma membrane proteins extracted from HUVECs or SVECs as described before (Shen et al., 2017). Following five washes, bound proteins were eluted in polyacrylamide gel electrophoresis (PAGE)-gel loading buffer and denatured by boiling for 10 min. After separation in 12% sodium dodecyl sulfate–PAGE (SDS-PAGE) gel and transferred into nitrocellulose membrane, hypothetical FlgE-interacting proteins were probed with a commercial anti-ATP5B antibody (Abcam, Cambridge, MA), or with anti-His antibodies (ProteinTech Group, Chicago, IL) as loading control.

### Confocal Microscopy Assay of the Distribution of FlgE/Mutant FlgE and Ectopic ATP5B on Human Umbilical Vein Endothelial Cells and SV40-Transformed Endothelial Cells of Mouse

HUVECs and SVECs cultured on glass coverslip were incubated with His-FlgE/His-FlgEM (10 μg/ml) and anti-ATP5B antibodies (5 μg/ml) for 1 h. The cells were fixed with 4% paraformaldehyde (PFA) for 20 min and blocked with 3% bovine serum albumin (BSA) for 30 min. FlgE was stained green with primary mouse anti-6× His antibodies (0.6 μg/ml, ProteinTech Group) at 4°C overnight and secondary fluorescein isothiocyanate (FITC)–anti-mouse IgG (eBioscience, San Diego, CA) for 1 h. EctoATP5B was stained red using phycoerythrin (PE)–anti-rabbit IgG (BioLegend, San Diego, CA) for 1 h. The images were observed under a confocal fluorescence microscope (Leica, Wetzlar, Germany).

### Flow Cytometry Assays of Proteins FlgE/Mutant FlgE or Peptides Pc-B/Pc-F Binding to Ectopic ATP5B on Endothelial Cells

HUVECs and SVECs (2 × 10^5^ cells/200 μl) were resuspended in ice-cold phosphate-buffered saline (PBS) (2% FBS) and directly incubated with different reagents (i.e., recombinant FlgE or FlgEM at 10 μg/ml, or FITC-Pc-B or FITC-Pc-F at 50 μM) for 40 min at 4°C. In blocking experiment settings, VECs were pretreated with anti-ATP5B antibodies (50 μg/ml), FlgE (10 μg/ml), FlgEM (10 μg/ml), or Pc-B/Pc-F peptides (50 μM) for 40 min before the abovementioned incubation. After two washes with staining buffer, cells were stained with FITC-conjugated anti-His antibodies (0.6 μg/ml, Abcam) or PE-conjugated anti-rabbit IgG (0.6 μg/ml, BioLegend) for 30 min at 4°C in the dark. Cells directly stained with FITC-anti-mouse IgG antibodies or PE-anti-rabbit IgG antibodies were used as controls. All samples were run in a FACScan flow cytometer (Beckman Coulter, Brea, CA), and data were analyzed using FlowJo 10 (Tree Star, Ashland, OR). Mean fluorescence intensity (MEI) of cells in each sample was used directly for comparison.

### ELISA Detection of Peptides Pc-B/Pc-F Binding to Ectopic ATP5B Proteins Prepared From Endothelial Cells or to Recombinant ATP5B Analogs

Luminescence test plates were coated with anti-ATP5B antibodies (1 μg/ml, 100 μl/well) overnight and blocked with 5% BSA at 37°C for 2 h. Membrane proteins were then added (5 μg/100 μl/well) for 40 min. After washing, recombinant FlgE proteins were added (as blockers) to 10 μg/100 μl. Then serial diluents of FITC-conjugated peptides Pc-B or Pc-F were added for 40 min; and after washing, the plates were read. In another setting, the plates were coated with a recombinant ATP5B analog consisting mainly of predicted surface epitope tandem (i.e., rATP5BesT proteins (Wang et al., 2019), 5 μg/100 μl) and used as above.

### Effects of FlgE/Mutant FlgEM Proteins on Vascular Endothelial Cell Functions or Behavior *In Vitro*

*In vitro* effects of FlgE or FlgEM proteins on cultured HUVECs or SVECs were measured using several different routine protocols and readouts, respectively, including ATP production, proliferation, survival (apoptosis and lactate dehydrogenase release), migration, and tube-like structure formation. Different concentrations were utilized based on pilot studies. In some assays, the cells were pretreated with anti-ATP5B antibodies or peptides Pc-B/Pc-F as detailed in **Supplementary Material/Methods**.

### Measurement of Permeability of Cultured Endothelial Monolayer *In Vitro*

Permeability of endothelial monolayers *in vitro* was determined by FITC-conjugated dextran (FITC-dextran) and transendothelial migration of neutrophils. Briefly, HUVECs or SVECs were seeded on collagen-coated upper chambers (2 × 10^5^ cells/200 μ/well) of transwell inserts (diameter 6.5 mm, pore size 0.4 μm, polycarbonate membrane, Costar); and lower chambers were supplemented with 0.5 ml of culture medium. The cells were cultured for 24 h to allow monolayer form. After pretreatment with different doses of anti-ATP5B antibodies or peptides for 1 h, cells were treated with 20 μg/ml of FlgE or FlgEM for 24 h. Then, 200 μl of FITC-dextran solution (0.5 mg/ml, 40 kDa, Sigma) was added into the upper chambers; and the culture was continued for 2 h at 37°C. Samples were collected from lower chambers, and leaked fluorescence was measured using a fluorescent plate reader. In another experimental setting, a filter with 5.0-μm pore size was utilized for collagen coating, HUVEC growing, and anti-ATP5B antibody treatment. Then neutrophils obtained from peripheral blood of healthy donors were added to the upper chambers (10^5^ neutrophils in 100 μl of medium), and FlgE or FlgEM proteins (20 μg/ml) were loaded to the lower chambers (or not). One hour later, cells that migrated into the lower chambers were countered using a Coulter Counter (BodBoge, Shenzhen, China).

### Detection of Cellular F-Actin Organization in Human Umbilical Vein Endothelial Cells

HUVECs were seeded onto glass coverslips placed in 24-well plates till growth achieved confluence. Anti-ATP5B antibodies (50 μg/ml) were added for 1 h, followed by FlgE or FlgEM (20 μg/ml) challenge for 2 h. Cells were washed with pre-warmed PBS and fixed with 4% PFA for 20 min at 37°C. The cells were then permeabilized with 0.5% Triton X-100 for 15 min and blocked for 1 h with 3% BSA in PBS at room temperature. For F-actin staining, 5 μg/ml of FITC-labeled phalloidin (Sigma) was added for 40 min. Nuclei were counterstained with DAPI. After washing, the coverslips were observed with a fluorescence microscope (Leica), and images were captured.

### Real-Time PCR Detection of FlgE-Modulated Gene Transcription

HUVECs (1 × 10^5^ cells) grown in 24-well plates were pretreated with or without 50 μg/ml of anti-ATP5B antibodies for 1 h, followed by incubation with 20 μg/ml of FlgE or FlgEM for 24 h. In another experiment, HUVECs were directly treated with different doses of Pc-B or Pc-F peptides (0, 1, 10, and 100 nM) for 24 h. Total RNA was extracted with a RNAiso Plus reagent (Takara, Shiga, Japan); reverse transcribed using Rever Tra Ace^®^ qPCR RT Kit (Toyobo, Katata Otsu Shiga, Japan); and subjected to RT-PCR in a Biosystems 7500 Real-Time PCR System (Applied Biosystems, Foster, CA) in 10 μl of SYBR^®^ Premix Ex Taq™ II Kit (Takara). Sequences of PCR primers of target genes are given in **Table S1**. PCR conditions were as follows: 95°C for 15 s, 40 cycles of 95°C for 5 s, and 60°C for 35 s. Ct of each reaction was obtained by SDS System Software of Applied Biosystems, and the statistical methods were detailed as before (Li et al., 2019).

### Western Blotting Assay

HUVECs were stimulated with 20 μg/ml of FlgE or FlgEM for desired time lengths or pretreated with 50 μg/ml of anti-ATP5B antibodies for 1 h. Total proteins were extracted and subjected to 12% SDS-PAGE resolution, followed by transferring onto nitrocellulose membrane (Beyotime, Shanghai, China) for immunoblot analysis. The primary antibodies used were as follows: anti-VE-cadherin antibodies (1 μg/ml, Abcam), anti-phospho-VE-cadherin (Tyr731) antibodies (1 μg/ml, Affbiotech, Shanghai, China), anti-occludin antibodies (1 μg/ml, Abcam), anti-claudin-1 antibodies (1 μg/ml, Abcam), and anti-GAPDH antibodies (1 μg/ml, ABsin, Shanghai, China). Primary probing was carried out overnight at 4°C and, after washing, was incubated with horseradish peroxidase (HRP)-conjugated anti-rabbit IgG antibodies (1:2,000, Cell Signaling Technology, Beverly, MA). The blots were detected using Enhanced Chemiluminescence (ECL) Amersham Western Blotting Detection Reagents (GE Healthcare).

### Measurement of Microvascular Permeability *In Vivo*

**Figure 1:**
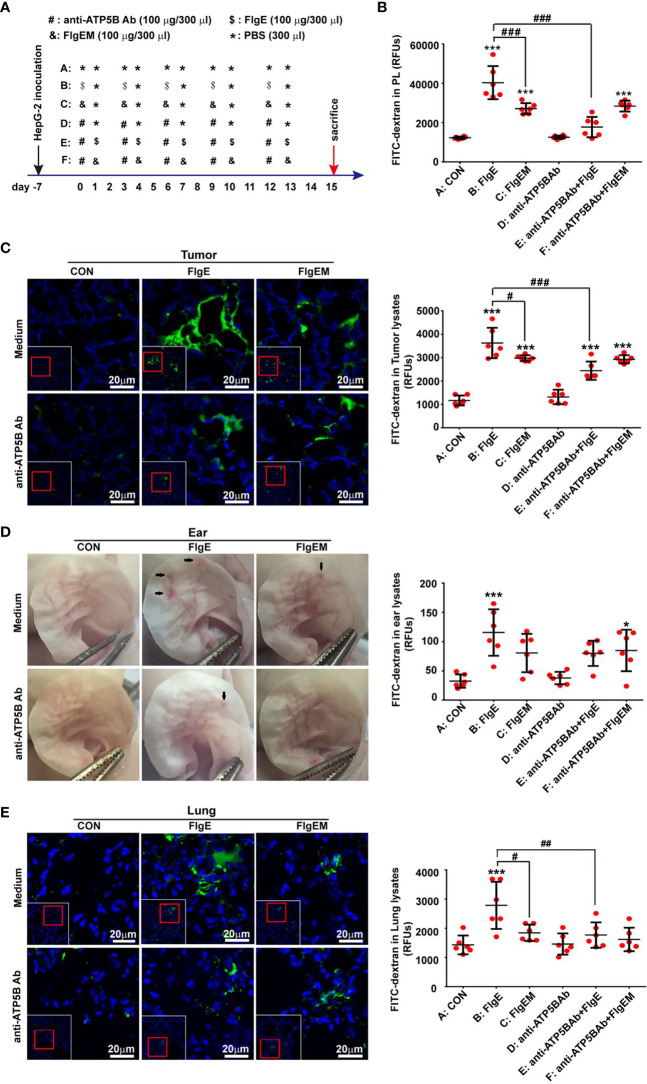
FlgE-induced vascular permeability in tumor-bearing animal models was alleviated by anti-ATP5B antibodies. Thirty-six 4-week-old nude mice were inoculated with 10^7^ HepG2 cells subcutaneously at the right anterior armpits. A week later, the mice were divided into six groups and received different treatments **(A)**. On day 15, images of ears were captured before receiving 0.2 ml of FITC-dextran (2.5 mg/ml) *via* tail vein. Thirty minutes later, mice were anesthetized, and cardiac perfusion was performed with 20 ml of PBS. The abdominal cavity was lavaged with 1 ml of PBS to generate peritoneal lavage fluid (PL) **(B)**. Tumor mass **(C)**, ears **(D)**, and lungs **(E)** were harvested. Tissues were either homogenized directly for fluorescence measurement as in abdominal ascites **(B–E)** or fixed and subjected to confocal microscopy examination after nuclear labelling with DAPI, where leaked dextran was seen as green **(C, E)**. Arrows in **(D)** indicate leakages seen by the naked eyes. Scale bar, 20 μm. Representative images are shown for either histological sections or gross ears. Quantification of leaked FITC-dextran in lungs or tumors tissues was expressed as fluorescence units per micrograms of homogenates. Data are representative of duplicate independent experiments with similar results. *p < 0.05, ***p < 0.001 *vs.* control (CON). ^#^*p* < 0.05, ^##^*p* < 0.01, ^###^*p* < 0.001 *vs.* FlgE, all by one-way ANOVA test. HUVECs, human umbilical vein endothelial cells; SVECs, SV40-transformed endothelial cells of mouse; VECs, vascular endothelial cells; FITC, fluorescein isothiocyanate; PBS, phosphate-buffered saline.

A murine tumor model was set up, and tumor mass as well as other tissues were measured for microvascular permeability *in vivo* utilizing FITC-dextran as tracer as described (Tichet et al., 2015) with modification. In brief, male nude mice at 4 weeks old were injected subcutaneously with 10^7^ HepG2 cells at the right anterior armpit. Seven days later, some of the animals were given 100 μg of anti-ATP5B antibodies or the same volume of PBS *via* intraperitoneal injection. Twenty four hours later, PBS (300 μl), FlgE (100 μg/300 μl), or FlgEM (100 μg/300 μl) was similarly administered. Anti-ATP5B and proteins treatments were repeated every third day for a total of four times (refer to **Figure 1A** for schematic illustration). On day 15, the right ear of each mouse was photographed, and each mouse received 0.2 ml of 2.5 mg/ml FITC-dextran *via* tail vein. Thirty minutes later, mice were anesthetized, and cardiac perfusion was performed with 20 ml of PBS to remove dextran from the vessels. Then abdominal cavity was rinsed with 1 ml of PBS. Lung and tumor tissues were harvested and sectioned. A portion was homogenized directly for fluorescence measurement or fixed with 4% PFA overnight, embedded in optimal cutting temperature (OCT), and cut into 4-μm sections for confocal microscopy examination. Images were captured by confocal microscope. FITC-dextran extravasation in tissue homogenate or abdominal ascites was measured by fluorescence plate reader (BioTek, Winooski, VT).

### Structural Modeling of the FlgE–ATP5B Complex

The crystal 3D structure of FlgE was primarily modeled by this team (Shen et al., 2017) and refined here by a 100-ns molecular dynamics (MD) simulation with CHARMM force field (Huang and MacKerell, 2013) using NAMD (Phillips et al., 2005). To define proposed interactions between FlgE and ATP5B molecules, we scanned the surface of ATP synthase with the sequences of epitopes B and F for potential binding sites by FlgE proteins. This was done by using the PEP-SiteFinder (Saladin et al., 2014). The top 10 poses were checked to identify the most likely binding sites. With these sites as restriction, structural modeling was performed using a protein–protein docking tool, Zdock (Pierce et al., 2014). Then the probable FlgE–ATP5B complex model was further refined or equilibrated by a 10-ns MD simulation as above, and the types and strength of the interaction forces between the two components in the complex were determined.

### Immunofluorescence of VE-Cadherin Distribution

HUVECs grown on coverslips were treated with FlgE or FlgEM (20 μg/ml) for 24 h. Cells were fixed with 100% methanol and permeabilized with 0.5% Triton X-100. VE-cadherin was stained using anti-VE-cadherin antibodies (1 μg/ml, Abcam) at 4°C overnight, followed by incubation with Alexa Fluor 594-labeled anti-rabbit IgG (1 μg/ml, Abcam) for 1 h at room temperature. DAPI (1 μg/ml, Beyotime) was used to stain the cell nuclei. Images were captured with a fluorescence microscope (Leica).

### Staining and Measurement of PAO1 Bacteria Bound to Human Umbilical Vein Endothelial Cells *In Vitro*

To mimic actual vascular *Pseudomonas* infection, an *in vitro* coculture of HUVEC and PAO1 was modified from a previous report (Elhenawy et al., 2019). In brief, HUVECs were grown on glass coverslips to confluency. PAO1 bacteria were washed three times with PBS and resuspended in RPMI-1640 at 5 × 10^8^ CFU/ml and added to HUVEC culture. After incubation for 1 h at 37°C, the coverslip with the HUVECs were washed with PBS for three times and fixed with 4% PFA for 20 min. To stain intracellular components such as mitochondrial ATP5B and if there were intracellular FlgE, the fixed cells were permeabilized with 0.25% Triton X-100 for 10 min followed by blocking with 3% BSA for 0.5 h. The customized primary polyclonal rabbit anti-FlgE and mouse anti-ATP5B antibodies (Abcam) were used at 1 μg/ml overnight at 4°C. After washes, Alexa-Flour 555 conjugated anti-rabbit IgG and Alexa-Fluor 488 conjugated anti-mouse IgG (both from Beyotime Institute of Biotechnology, Shanghai, China) were added onto coverslip for 1 h. Images were captured with a confocal fluorescence microscope (Leica).

To quantify the PAO1–HUVEC adhesion and to check the possibility for targeting this interaction, HUVECs were grown in 24-well plate till confluence, washed three times, and incubated with 3% BSA (Sigma). Anti-ATP5B antibody was added to 100 μg/ml or Pc-B/Pc-F peptides to 100 μM for 1 h. Then 500 μl of aliquots of wild-type PAO1 suspension (1 × 10^7^ CFU/ml in RPMI-1640 culture medium) was added to each well at 37°C for 1 h. Then the culture medium containing unbound bacteria was discarded, and the HUVECs were washed with PBS for three times to remove non-associated bacteria. Cells in each well were lysed with a lysis buffer (0.25% trypsin, 0.02% EDTA, 0.01% Triton X-100) for 10 min. The lysates were diluted by 10^5^-fold, spread on Luria broth (LB) agar plates, and cultured at 37°C for 20 h. The resulting colonies were counted under microscope. At least three wells were used for each experiment. In parallel setting, mutant PAO1 strains with mutations on sites B and F of FlgE proteins, namely, PAO1/*flgEΔBmF*) and PAO1/*flgEmF* (Shen et al., 2017), were used similarly and compared with wild-type PAO1 strain.

### Statistical Analysis

The setting of duplicates and repetition of experiments were detailed in the figure legends. The average of numerical data was expressed as mean ± SD. All results were analyzed with Graph Pad Software version 7.0, and applicable methods are specified in the figure legends. When one-way ANOVA showed differences among groups and to avoid class I errors, Duncan’s new multiple range method was further used to compare the data between each group. When two-way ANOVA suggested significant interaction, between-subjects effects and pairwise comparison were performed with Bonferroni’s or Tukey’s assay among the groups.

## Results

### FlgE Proteins Bound Ectopic ATP5B on Endothelial Cell Surface

**Figure 2:**
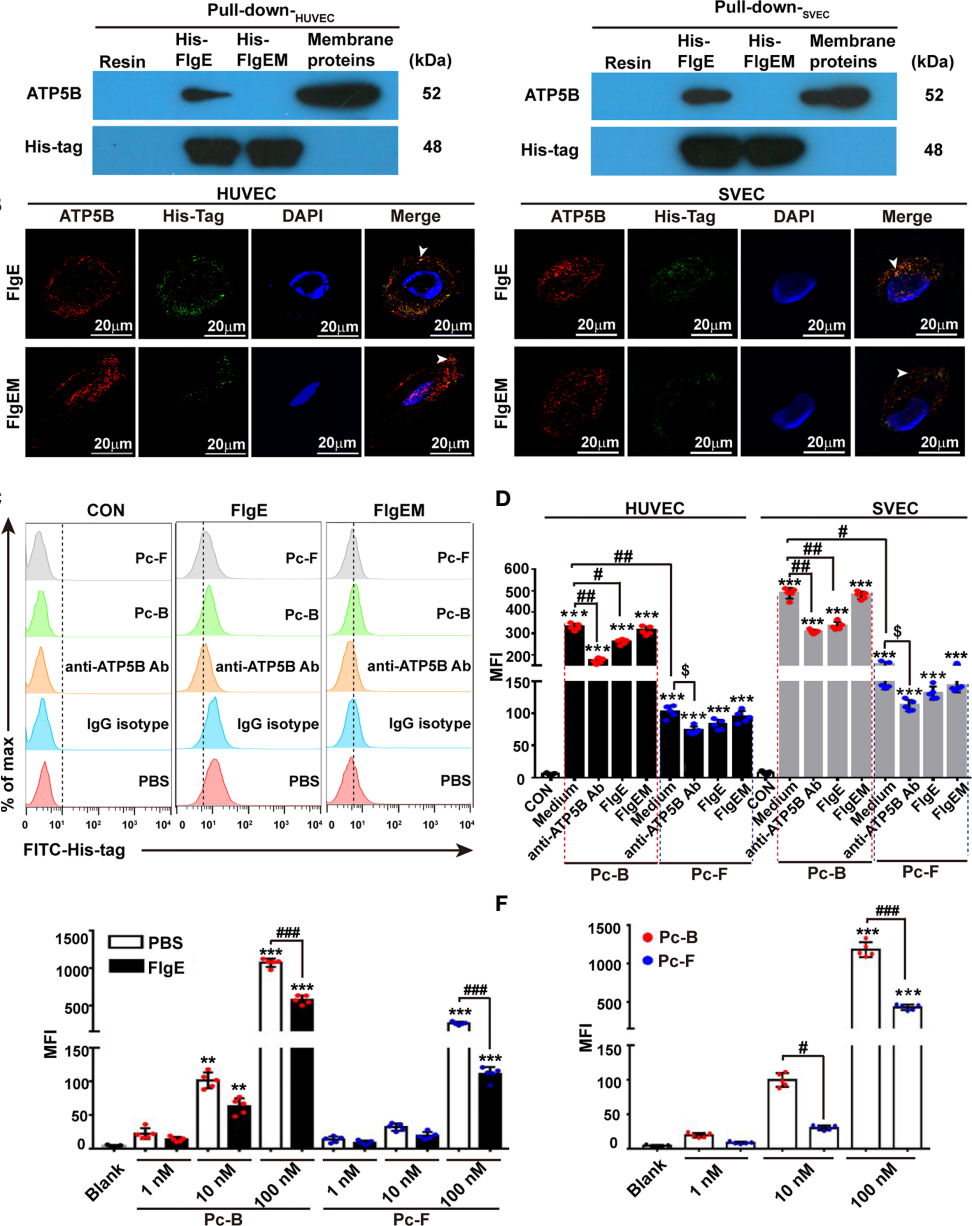
Binding of recombinant FlgE proteins onto ectopic ATP5B on endothelial cells. **(A)** Pull-down analysis demonstrated affinity between recombinant FlgE proteins and ATP5B in membrane lysates prepared from HUVEC or SVECs. Membrane proteins of VECs were incubated with either His-FlgE- or His-FlgEM-conjugated Ni-NTA beads (lanes 2 and 3 from the left) or nude Ni-NTA resin beads (far left lanes), and recovered proteins from the beads were subjected to routine immunoblotting with anti-ATP5B antibodies or anti-6× His-tag antibodies. Equivalent amount of starting membrane lysates was run at side as positive control of ATP5B proteins (far right lanes). **(B)** Surface localization of FlgE/FlgEM and ectoATP5B on HUVECs and SVECs. HUVECs and SVECs grown on glass coverslip were incubated with FlgE (10 μg/ml) and anti-ATP5B antibodies (50 μg/ml) for 1 h The cells were fixed with 4% PFA and blocked with 3% BSA. FlgE was stained with primary mouse anti-6× His-tag antibodies and FITC-anti-mouse IgG (green). EctoATP5B was stained *via* PE-anti-rabbit IgG (red). White arrowheads pointed at colocalization of FlgE and ectoATP5B on HUVECs and SVECs. **(C)** FlgE binding to VECs was inhibited by anti-ATP5B antibodies or Pc-B/Pc-F peptides. HUVEC and SVECs were preincubated for 1 h with anti-ATP5B antibodies (50 μg/ml), isotype IgG, or Pc-B/Pc-F peptides (50 μM) followed by incubation with 10 μg/ml of His-FlgE or His-FlgEM for 40 min. Then the cells were stained with FITC-conjugated anti-6× His antibodies (0.6 μg/ml) and measured by flow cytometry. **(D)** Binding Pc-B/Pc-F peptides on VECs were inhibited by competitive anti-ATP5B antibodies or recombinant FlgE proteins. HUVECs and SVECs were treated with either anti-ATP5B antibodies (50 μg/ml) or FlgE (10 μg/ml), followed by addition of FITC-Pc-B or FITC-Pc-F peptides (50 μM) and sampling in a flow cytometer. Mean fluorescence intensity (MFI) was calculated for all cells. ***p < 0.001 *vs.* CON. ^#^*p* < 0.05, ^##^*p* < 0.01 *vs.* Pc-B. ^$^*p* < 0.05 *vs.* Pc-F alone without antibody. **(E)** Binding of Pc-B/Pc-F peptides on membrane ATP5B proteins was dose-dependent and inhibited by recombinant FlgE proteins. Luminescence test plates were coated with anti-ATP5B antibodies (1 μg/ml) at 4°C overnight, followed by incubation with plasma membrane proteins (5 μg/100 μl, obtained from HUVECs). After blocking with 10 μg/100 μl of FlgE or not (PBS), FITC-Pc-B or FITC-Pc-F peptides were added into the wells to indicate concentrations. After incubation at 37°C for 40 min and three washes, the plates were read for fluorescence intensity. **p < 0.01, ***p < 0.001 *vs.* Blank. ^###^*p* < 0.001 *vs.* PBS. **(F)** ELISA of FITC-Pc-B and FITC-Pc-F peptides binding to ATP5B analog rATP5BesT. rATP5BesT proteins were used to coat ELISA plates at 5 μg/ml overnight, FITC-Pc-B and FITC-Pc-F peptides were added to different concentrations, and bound peptides were measured as above. ***p < 0.001 *vs.* Blank. ^#^*p* < 0.05, ^###^*p* < 0.001 *vs.* Pc-B. Representative results of three repeats are exhibited. Individual data are shown as dots, while mean ± SD as columns with bars. Two-way ANOVA was performed for statistical comparison. HUVECs, human umbilical vein endothelial cells; SVECs, SV40-transformed endothelial cells of mouse; VECs, vascular endothelial cells; FlgEM, mutant FlgE; PFA, paraformaldehyde; BSA, bovine serum albumin; FITC, fluorescein isothiocyanate; PE, phycoerythrin; PBS, phosphate-buffered saline.

We first checked whether the hypothetical interactions between FlgE and ATP5B proteins would actually occur in VECs. Affinity chromatography with membrane extractions of human HUVECs or murine SVECs onto FlgE proteins (or FlgEM as control) showed that recombinant FlgE proteins bound to and pulled down ATP5B proteins from those membrane extractions (**Figure 2A**). Binding of FlgE proteins to intact HUVEC and SVECs was also confirmed with immunostaining (**Figure 2B**). Flow cytometry showed that bindings of FlgE proteins with HUVECs were inhibited by pretreatment of cells with anti-ATP5B antibodies (**Figure 2C**), suggesting that FlgE interacted with ectoATP5B directly. In previous works, we identified two epitopes (i.e., sites B and F) of FlgE that were essential for immunostimulating potency of FlgE proteins. To determine whether these sites were also pivotal for FlgE–ectoATP5B interaction, two synthetic peptides corresponding to these two epitopes, Pc-B and Pc-F, were tested for their potential binding with HUVECs or SVECs or with immobilized cellular membrane extract. It was found that both Pc-B and Pc-F peptides bound the cells (**Figure 2D**) or cellular proteins (**Figure 2E**), and the bindings were partially impaired by presence of anti-ATP5B antibodies or FlgE proteins (**Figures 2D, E**). *Vice versa*, Pc-B and Pc-F peptides partially blocked FlgE binding to HUVECs (**Figure 2C**). In another ELISA, an ATP5B analog composed of a tandem of main predicted B cell-recognized immunological epitopes (rATP5BesT) produced in this lab (Wang et al., 2019) was tested for its binding with Pc-B/Pc-F peptides, and it was found that both peptides bound rATP5BesT in a dose-dependent manner (**Figure 2F**). In all these binding-based assays, FlgEM proteins showed much less or deficient potency than did FlgE proteins (**Figures 2A–D**), or Pc-F demonstrated less affinity than Pc-B (**Figures 2D, F**).

### Structural Modeling Supported Interactions Between FlgE and ATP5B Molecules

**Figure 3:**
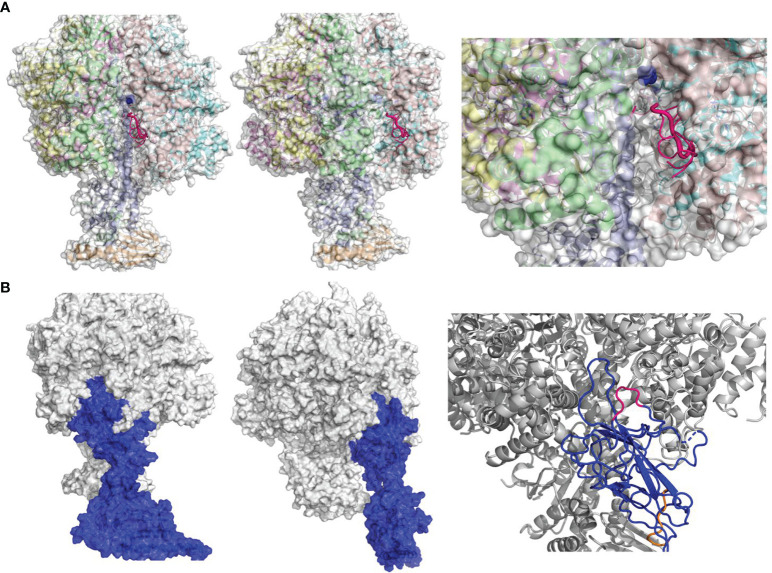
Prediction of FlgE–ATP5B interacting mode by structural modeling. **(A)** Identification of potential binding site(s) on the surface of ATP synthase (PDB 1e79) for Pc-B and Pc-F peptides. Six or 8 of the top 10 best poses for B and F respectively were located onto a same groove on the beta subunit (chain E, 1e79_E) shown in front (left panel), side (middle), and close-up views (right). Only the peptides of B (in hot pink) are illustrated. The binding site involved residues 159–162 of chain E **(B)** The docking model of FlgE–ATP5B complex in front, side, and close-up views. FlgE is illustrated in blue and ATP5B in mixed color, while site B and site F are shown in hot pink and orange, respectively, in the close-up view (right).

The above data collectively demonstrated that FlgE proteins could directly bind ectoATP5B on either human or murine VECs, and this binding involved epitopes B and F at FlgE side. In support of this notion obtained from functional investigations, structural analysis of Protein Data Bank (PDB) data of FlgE and ATP5B demonstrated that peptides Pc-B/Pc-F most likely bind to a same groove on the surface of ATP5B (**Figure 3A**). In brief, when Pc-B and Pc-F sequences were screened randomly for potential binding sites on ATP synthase complex (PDB, 1e79_E), six and eight of the top 10 best poses for B and F, respectively, were located onto a groove on the β subunit chain E in the 3D model of ATP synthase, strongly suggesting that this site was the top one candidate for epitope B or F binding (**Figure 3A**). By restricting the whole FlgE molecule to this groove, FlgE–ATB5B docking was carried out, and the best scored FlgE–ATB5B model was furthered equilibrated by a 10-ns MD simulation. As shown in **Figure 3B**, FlgE utilized site B to anchor onto the groove, while site F lie beside the groove (**Figure 3B**). Further calculation and analysis of the non-bonded interactions between FlgE and ATP5B suggested that both electrostatic interactions and Van der Waals force contributed to the binding of FlgE on ATP5B (**Supplementary Table S2**), and the total forces were of the same order with that reported for SARS-CoV-2 and ACE2 (Spinello et al., 2020).

### Binding of FlgE to Ectopic ATP5B Modulated Endothelial Cell Behaviors

**Figure 4:**
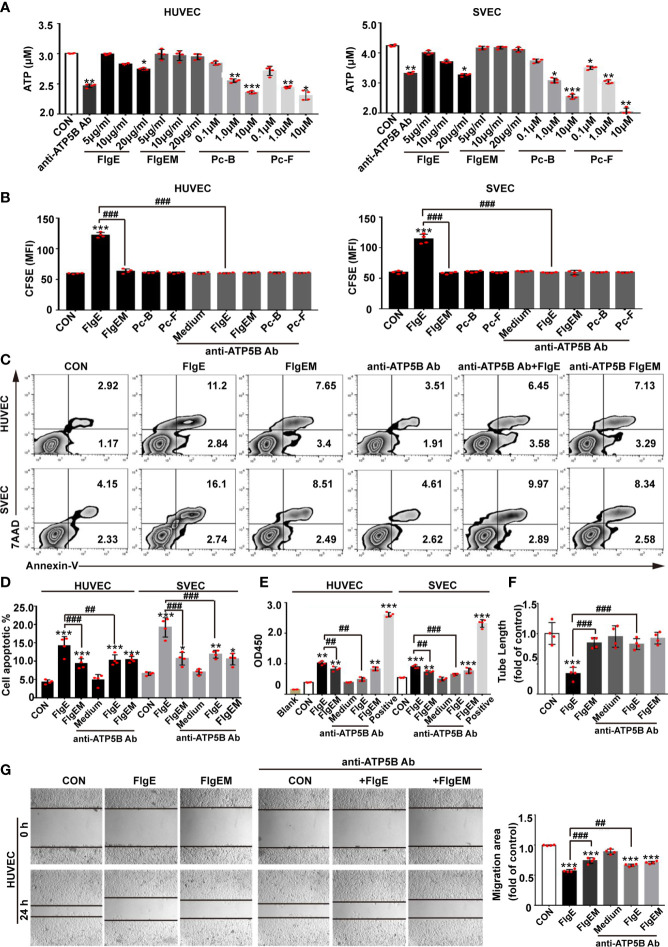
Modulation of VECs behaviors by recombinant FlgE proteins was sensitive to anti-ATP5B interference. **(A)** Production of surface ATP catalyzed by ectoATP synthase. VECs were pretreated with anti-ATP5B antibodies (50 μg/ml), peptides (0.1, 1, and 10 μM), or FlgE/FlgEM proteins (5, 10, and 20 μg/ml) for 1 h, and ADP was added to 100 μM with 2 mM of Mg^2+^ and 10 mM of P_i_. Medium was harvested 15 s later; and ATP contents (as reflected by relative luminescence unit (RLU)) were measured using an ATP assay kit. *p < 0.05; **p < 0.01; ***p < 0.001 *vs.* control (CON), all by Dunnett’s test. **(B–E)** VECs were incubated with FlgE or FlgEM (both 20 μg/ml) and peptides Pc-B/Pc-F (both 100 μM) in the presence of anti-ATP5B antibodies (50 μg/ml) or not, for the indicated time length. Cell proliferation was determined by CFSE assay at 48 h, with higher mean fluorescence intensity (MFI) indicating less proliferation of the whole cell population **(B)**, or cell death was measured at 4 h with flow cytometry (**C**, with annexin-V staining for cell membrane change and 7AAD staining for nuclear DNA; **D**, statistics of samples in panel **C**) or lactate dehydrogenase releasing assay (**E**, as reflected by OD490; “Positive” represents a lactate dehydrogenase-releasing reagent supplied in the kit). **(F)** HUVECs were seeded into Matrigel-coated 96-well plates (2 × 10^4^ cells/well) with different reagents (i.e., anti-ATP5B antibody 50 μg/ml, FlgE or FlgEM 10 μg/ml, or a combination). After 4 h of incubation, tube formations were observed under a microscope. Capillary-like tubes were traced, and total tube length of each group was calculated using ImageJ software. **(G)** HUVECs were grown in 24-well plates to confluence, switched to serum-free culture medium, and pretreated with anti-ATP5B antibodies (50 μg/ml) for 1 h; and then a scratch was made across the cell layer using a yellow tip. The cultures were rinsed, FlgE or FlgEM was added at 20 μg/ml in serum-free medium, the culture was continued for 8 h Images were captured under a microscope at the same sites of the scratches before and after the last 8-h incubation (left panel), and migration was quantified with ImageJ software. The migration potency under treatment was calculated as the ratio of migration distance to that in control groups (right panel). All experiments were repeated three times, and representative ones were shown. *p < 0.05, **p < 0.01, ***p < 0.001 *vs.* control (CON). ^##^*p* < 0.01, ^###^*p* < 0.001 *vs.* FlgE, by two-way ANOVA test. VECs, vascular endothelial cells; CFSE, carboxyfluorescein succinimidyl ester; HUVECs, human umbilical vein endothelial cells.

It was not fully understood yet how ectopic ATP synthase in different cells responded to different types of ligands (e.g., angiostatin or Apo A-I). To get an idea of whether or how FlgE would affect VECs properties, the effects of FlgE proteins or peptides Pc-B/Pc-F on VEC behaviors were studied. When the main biochemical activity of ATP synthase (i.e., to catalyze extracellular ATP synthesis) was looked at, FlgE proteins as well as peptides Pc-B/Pc-F and anti-ATP5B antibodies all inhibited extracellular ATP production by HUVECs or SVECs in a dose-dependent manner (**Figure 4A**). However, only FlgE proteins and not peptides Pc-B/Pc-F inhibited the proliferation of HUVECs or SVECs (**Figure 4B**). Similarly, FlgE increased apoptosis of both cell lines as measured using fluorescence-activated cell sorting (FACS) (**Figures 4C, D**) or lactate dehydrogenase release (**Figure 4E**). FlgE proteins also inhibited tube-like structure formation (**Figure 4F**) and migration (**Figure 4G**) of HUVECs (SVECs not detected). In all these assays, FlgEM manifested decreased effects compared with FlgE. The effects of FlgE were abrogated by pretreatment with anti-ATP5B antibodies (50 μg/ml), while anti-ATP5B antibodies of themselves alone at this concentration had no effect on VEC behaviors (**Figures 4B–G**). In short, FlgE proteins modulated VEC behaviors by binding to ectoATP5B, and epitopes B and F of FlgE were involved in such binding.

### FlgE Proteins Induced Interleukins and Matrix Metalloproteinase Transcription in Endothelial Cells in Ectopic ATP5B-Dependent or Ectopic ATP5B-Independent Manner, Respectively

**Figure 5:**
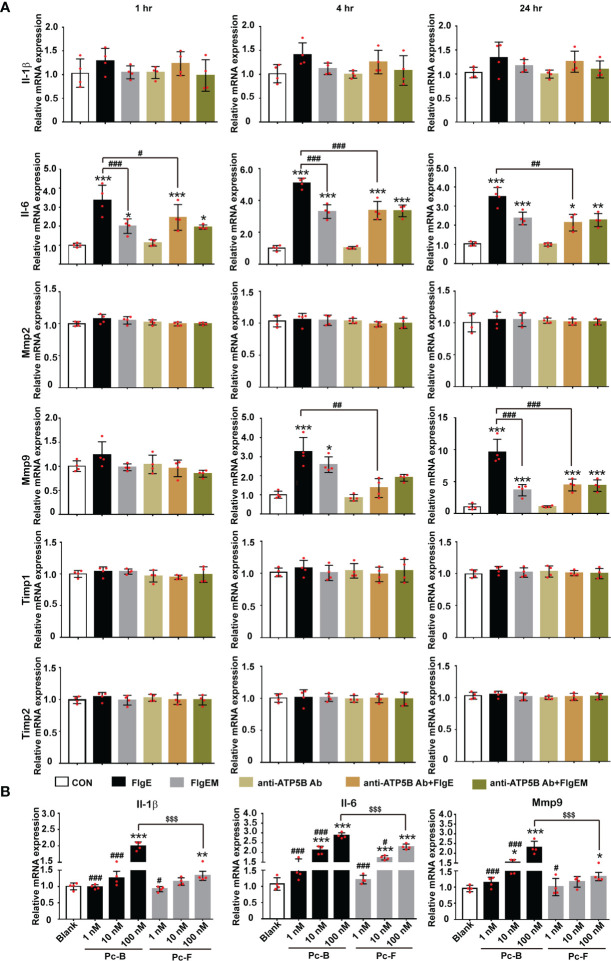
FlgE-induced expression increase of IL-1, IL-6, and Mmp9 in HUVECs. HUVECs were pretreated with anti-ATP5B antibodies (50 μg/ml) for 1 h; and then FlgE or FlgEM was added to 20 μg/ml for different time lengths (1, 4, and 24 h) **(A)** or subjected to treatment with different concentrations of Pc-B or Pc-F peptides for 4 h **(B)**. Total RNA was extracted and subjected to reverse transcription and PCR. Expression level of each gene in control group was arbitrarily set at 1.0, and relative levels in all treatments were obtained by calculation with Ct. *p < 0.05, **p < 0.01; ***p < 0.001 *vs.* control (CON). ^#^*p* < 0.05, ^##^*p* < 0.01, ^###^*p* < 0.001 *vs.* FlgE **(A)** or 100 nM of the same treatment **(B)**. ^$$$^*p* < 0.001 *vs.* Pc-B **(B)**, all by two-way ANOVA test. HUVECs, human umbilical vein endothelial cells.

Proinflammatory or immunostimulating efficacy of FlgE proteins was first reported in human epithelial cells and murine lungs (Shen et al., 2017; Li et al., 2019). Here, we showed that when added in HUVECs culture, FlgE proteins also induced a proinflammatory response reflected by increased mRNA expression of IL-1β and IL-6 (**Figure 5A**). However, production of these two genes showed different dependence on ATP5B pathway. While FlgE-induced IL-6 mRNA increase was partially neutralized by anti-ATP5B antibodies, IL-1β mRNA increase was not, implying that FlgE-induced IL-1 expression should be mainly mediated by non-ATP5B pathways. Besides, matrix metalloproteinase (MMPs) and tissue inhibitor of metalloproteinases (TIMPs) had been known to contribute to various endothelial functions. Among the four member genes of this family detected herein (i.e., Mmp2, Mmp9, Timp1, and Timp2), only Mmp9 mRNA manifested response in HUVECs to FlgE stimulation, and this response was blocked by anti-ATP5B antibodies (**Figure 5A**). Again, FlgEM was less potent than FlgE in stimulating IL-1β/IL-6/Mmp9 mRNA expression (**Figure 5A**), implying that sites B and F were required for FlgE stimulation. In line with this, peptides Pc-B and Pc-F were able to stimulate expression of IL-1β/IL-6/Mmp9 in HUVECs in a dose-dependent manner (**Figure 5B**).

### FlgE Proteins Modulated Permeability and Cytoskeletal Rearrangement in Endothelial Cells *In Vitro via* Stimulating Ectopic ATP5B

**Figure 6:**
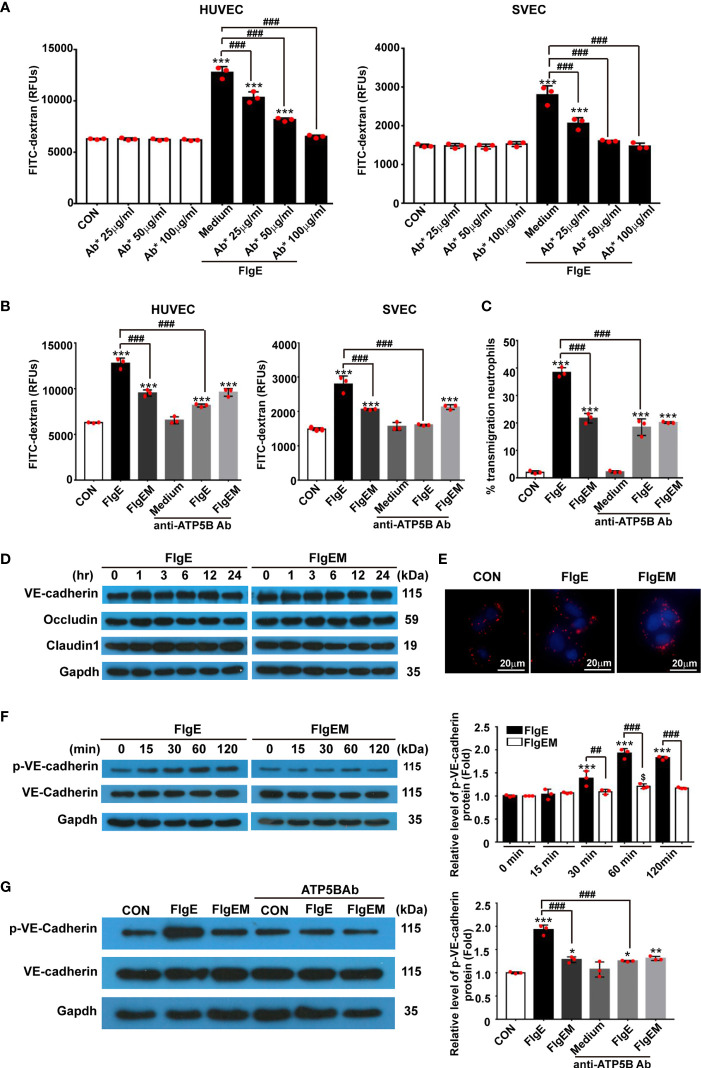
FlgE-induced hyperpermeability in EC monolayers was inhibited by anti-ATP5B antibodies and involved VE-cadherin phosphorylation. **(A–C)** Endothelial cells were cultured on collagen-coated filters for 24 h and then incubated with different concentrations of anti-ATP5B antibodies (25, 50, and 100 μg/ml) for 1 h followed by addition of FlgE or FlgEM (20 μg/ml). After 24 h, FITC-dextran (40 kDa) was added into the upper chamber (0.5 mg/ml) for 2 h, and the fluorescence in the lower chamber was measured in a fluorimeter **(A, B)**, or neutrophils isolated from human peripheral blood were added into the upper chambers and incubated for 1 h at 37°C, and the percentage of transmigrated neutrophils in the lower chambers was calculated **(C)**. **(D, F, G)** HUVECs, with or without 1-h pretreatment with anti-ATP5B antibodies (50 μg/ml), were treated with FlgE or FlgEM (20 μg/ml) for desired time length; and total proteins were subjected to blotting for VE-cadherin, Tyr731-phosphorylated VE-cadherin, occludin, or claudin-1, with GAPDH as reference. Resulting bands were analyzed with ImageJ software. **(E)** HUVECs were treated with FlgE or FlgEM (20 μg/ml) for 24 h, and the distribution of VE-cadherin is shown using confocal microscopy (VE-cadherin, red; DAPI, blue). Scale bars, 20 μm. Shown is one of the three repeats with similar results. *p < 0.05, **p < 0.01, ***p < 0.001, *vs.* CON. ^##^*p* < 0.01, ^###^*p* < 0.001 *vs.* FlgE by one-way **(A–C, G)** or two-way ANOVA **(F)** test. Mean ± SD was obtained from three duplicates in each experiment. HUVECs, human umbilical vein endothelial cells; EC, endothelial cell; FITC, fluorescein isothiocyanate.

To assess whether all the above effects of FlgE on VECs would affect the permeability of an endothelial layer, permeability of VEC layers to soluble FITC-dextran and granulate neutrophils were measured. FlgE increased the permeability of HUVEC or SVEC layers to FITC-dextran, and this effect was hampered by anti-ATP5B antibodies in a dose-dependent manner (**Figure 6A**). Again, FlgEM was less efficient in increasing VECs permeability, and its effect was not affected by the presence of anti-ATP5B antibodies (**Figure 6B**). In addition to FlgE- or FlgEM-induced hyperpermeability to soluble FITC-dextran, transendothelial migration of neutrophils across the VEC monolayer was also enhanced by FlgE proteins and, to lesser extent, FlgEM (**Figure 6C**). Again, anti-ATP5B antibodies inhibited hyperpermeability induced by FlgE, but not that induced by FlgEM (**Figure 6C**). To probe associated potential molecular mechanisms, protein levels of VE-cadherin, occludin, and claudin-1 in treated HUVECs were measured, but none showed any significant changes and the VE-cadherin protein was not endocytosed upon FlgE or FlgEM stimulation (**Figures 6D, E**). However, phosphorylation of VE-cadherin at Tyr731 was increased upon FlgE or, to lesser extent, FlgEM stimulation in time- and ATP5B-dependent manner (**Figures 6F, G**). Hypothetically, in line with the changes of cellular features including permeability induced by FlgE or FlgEM proteins, treatment of HUVECs with FlgE or FlgEM increased stress fiber formation as measured with immunofluorescence labeling of F-actin, and this activity of FlgE/FlgEM was countered by pretreatment of cells with anti-ATP5B antibodies (**Figure 1**).

### FlgE Proteins Induced Microvascular Hyperpermeability *In Vivo*

To further examine the effect of FlgE proteins on endothelial functions in animals, nude mice were inoculated subcutaneously with human HepG2 cells to establish a solid tumor model, which were subjected to different treatments (**Figure 1A**). Microvascular permeability in tumor mass as well as normal tissues such as the lungs, ears, and abdominal cavity was compared. After five 100-μg doses of FlgE proteins over a period of 15 days, leakage of intravenous FITC-dextran into the abdominal cavity (**Figure 1B**), proposedly *via* mesenteric vessels, into tumor mass (**Figure 1C**), into the ears (**Figure 1D**), and into the lungs (**Figure 1E**), was noted. FlgEM proteins of the same doses were weaker than FlgE proteins in this aspect. While FlgE-induced hyperpermeability was significantly inhibited by anti-ATP5B antibodies given 1 day before each FlgE dose, FlgEM-induced moderate leakage of FITC-dextran was not affected by anti-ATP5B antibody pretreatments (**Figures 1B–E**), indicating that overlapping but differential pathways were involved in FlgE- and FlgEM-induced microvessel damages.

### FlgE Proteins of Live PAO1 Contributed to PAO1–Human Umbilical Vein Endothelial Cell Interactions

To check whether the FlgE–ATP5B interactions observed by using recombinant/free/monomeric FlgE and cellular proteins would occur in an actual situation when *P. aeruginosa* bacteria enter the bloodstream, an *in vitro* infection model was set using living PAO1 bacteria and HUVECs. After 1 h of coculture, both FlgE and ATP5B were detectable on un-permeabilized cells, suggesting their distribution on the surface of PAO1-challenged HUVECs (**Figure 8A**, upper row). When the cells were permeabilized before immunostaining, the fluorescence signals for both FlgE and ATP5B (including from mitochondria) were much stronger (**Figure 8A**, bottom row), indicating the existence of FlgE in the cytoplasm of PAO1-challenged HUVECs. In a parallel quantitative assay, addition of Pc-B or Pc-F peptides in PAO1–HUVECs coculture system significantly inhibited wild-type PAO1 adhesion onto HUVECs as measured using colony-forming assay (**Figure 8B**). The decrease of adhesion induced by anti-ATP5B antibodies did not reach a threshold for statistical significance (e.g., *p* = 0.05). Furthermore, by using two PAO1 strains harboring mutations at both B and F sites (PAO1/*flgEΔBmF*) or at F site only (PAO1/*flgEmF*), it was shown that mutations of these two epitopes decreased PAO1 adhesion onto HUVECs when compared with wild-type PAO1 strain (**Figure 8B**).

## Discussion

**Figure 7:**
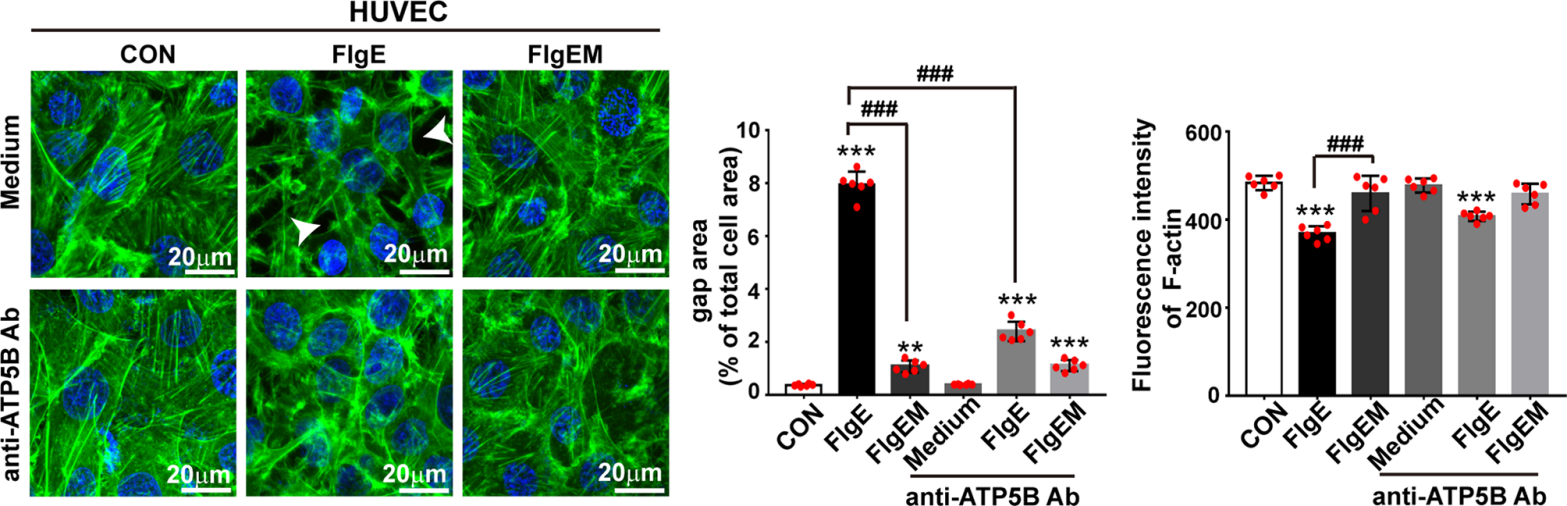
EctoATP5B contributed to FlgE-induced reorganization of F-actin cytoskeleton. HUVECs and SVECs grown on glass coverslip were preincubated with anti-ATP5B antibodies (50 μg/ml) for 1 h and then stimulated with 20 μg/ml of FlgE or FlgEM for 24 h. F-actin filaments of VECs were stained with FITC-phalloidin probes (50 μg/ml, green) and DAPI (blue), observed and captured with confocal microscopy, and analyzed with ImageJ. Scale bar, 20 μm. White arrow indicates gap formation among cells. The quantification of gap area and fluorescence intensity of F-actin was performed using ImageJ software. **p < 0.01, ***p < 0.001 *vs.* CON. ^###^*p* < 0.001 *vs.* FlgE by one-way ANOVA test. HUVECs, human umbilical vein endothelial cells; SVECs, SV40-transformed endothelial cells of mouse; VECs, vascular endothelial cells; FITC, fluorescein isothiocyanate.

The data described above demonstrated that FlgE proteins initiated responses in VECs upon binding to surface ectoATP5B. These findings advanced our knowledge about the immunological features of FlgE proteins as well as the roles of ectoATP5B in infection-related vascular disease. Firstly, the current study revealed more structural bases for the proposed FlgE immunostimulation. Both experimental binding/competition assays performed with FlgE or FlgEM proteins in combination with anti-ATP5B antibodies and protein structural modeling confirmed a sequence-dependent FlgE–ectoATP5B interaction, where the two epitopes (B and F) necessary for FlgE immunostimulation were among the “major” domains determining FlgE binding to ATP5B proteins. However, the fact that FlgEM was only partially disabled in various functional studies (**Figures 1**, **2**, **4**–**7**) suggested the existence of other or minor epitopes that also allow FlgE–ectoATP5B interaction to happen. Furthermore, the binding of FlgEM proteins with ATP5B seemingly requires an intact cellular membrane structure, so that in a reaction system like the one used for pull-down assay, the FlgEM–ATP5B affinity might be too weak for ATP5B to be pulled down by FlgEM (**Figure 2A**). Again, differential responses to anti-ATP5B antibody treatment in terms of producing IL-1 and IL-6 in HUVECs (**Figure 5**) implied that FlgE-induced production of these two cytokines involved ATP5B pathway differentially. Future studies should test if this observation is unique with FlgE of PAO1 only or applies with FlgE of other bacteria.

Secondly, by utilizing VEC cultures as well as vascular hyperpermeability models, the current study advanced our understanding of the pathogenesis of vascular disorders proposedly associated with acute or chronic infections. For example, without mentioning the acute-featured sepsis (Opitz et al., 2009; Khakpour et al., 2015; Salvador et al., 2016), the chronic-featured atherosclerosis had long been suspected to associate with unnoticed infections by certain types of microbes (Mayerl et al., 2006; Erridge et al., 2008; Calandrini et al., 2014; Xie et al., 2017; Raggi et al., 2018). Hansen et al. examined coronary thrombi obtained from 22 patients and identified a total of 55 different bacterial species, among which PA manifested the strongest correlation (Hansen et al., 2016). These clinical findings confirmed earlier experimental data of Turkay et al. that chronic infection of PA in rats promoted atherosclerosis development when the animals were on cholesterol diet (Turkay et al., 2004). At the molecular level, the current study added ectopic ATP synthase to the list of VECs receptors that sense and respond to pathogens, which ultimately result in hyperpermeability of VECs (Kim et al., 2016; Golovkine et al., 2017). Together with our previous report about caveolin-1 necessity in host response to FlgE (Shen et al., 2017), the current study confirmed the conjunctive action of caveolin-1 and ectoATP5B when responding to FlgE, just like in other processes involving VECs (Chi and Pizzo, 2006; Wang et al., 2006; Fu et al., 2011). Beside proving the colocalization of FlgE and ectoATP5B on the surface of PAO1-challenged HUVECs, we also showed that PAO1 entered HUVECs and interacted with intracellular FlgE. A limitation of this study was that though we demonstrated coexistence of FlgE and ATP5B in cytosol space of PAO1-infected HUVECs, we did not investigate whether these FlgE derived from engulfed PAO1 or newly generated PAO1 as a newly identified intracellular pathogen (Bernut et al., 2015). Or these FlgE signals were either for polymerized FlgE in flagella or for monomer FlgE secreted from live bacteria *via* the T3SS machinery (Garai et al., 2019). While the ATP5B in the cell plasma space was supposedly distributed along the inner membrane of mitochondria, a future study should check whether intracellular FlgE and ATP5B would be able to meet and interact with each other at all. If they do, the proposed theory of FlgE effect on ectopic ATP5B would also be applicable with those ectopic ATP5B proteins.

**Figure 8:**
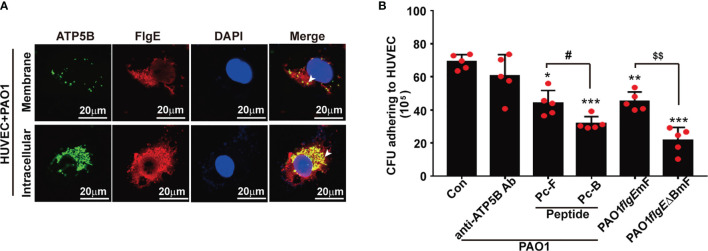
Observation and measurement of PAO1 adhesion onto and engulfment by HUVECs. **(A)** Immunofluorescence staining of FlgE and ATP5B protein in PAO1-infected HUVECs was performed after coculture of PAO1 with HUVECs for 1 h at 37°C. HUVECs were either permeabilized for intracellular staining or not permeabilized for staining surface components. FlgE was stained with Pc-FlgE Ab (1 μg/ml) in combination with anti-rabbit Alexa-Flour 555 (red), and ATP5B with murine anti-ATP5B antibody (1 μg/ml) in combination with anti-mouse Alexa-Flour 488 (green). White arrowheads point at colocalization of FlgE and ATP5B in HUVECs. **(B)** For measuring the effect of peptides or antibodies on PAO1–HUVEC adhesion, HUVEC monolayers grown in plates were incubated with anti-ATP5B antibodies (50 μg/ml) or peptides Pc-B/Pc-F (100 μM) for 1 h Then 500 μl of 10^8^/ml of wild-type PAO1 or mutant PAO1/*flgEmF* or PAO1/*flgE△BmF* strains was added and incubated for another 2 h After careful washing, the culture was harvested into lysis buffer, and the lysates were serially diluted and spread on LB agar plates for 20-h culture. The colonies were counted by the naked eyes. Data are shown as mean ± SD. *p < 0.05, **p < 0.01, ***p < 0.001 *vs.* Con. ^#^ or ^$^*p* < 0.05 as labeled. All by one-way ANOVA test. HUVECs, human umbilical vein endothelial cells; LB, Luria broth.

Thirdly, adhesion of bacteria to host cells poses a well-documented virulence, and flagella had been shown to initiate or contribute to such adhesion (Haiko and Westerlund-Wikstrom, 2013; Bruzaud et al., 2015). While in most situations concerning bacteria–host adhesions the “flagella” mainly refer to the filament part, the results of the current study support the role of the flagellar hook in such adhesions, partially *via* epitopes B and F (**Figure 8**) just like with FlgE immunostimulation activity (Shen et al., 2017; Li et al., 2019). The fact that Pc-B and Pc-F peptides blocked PAO1–HUVEC adhesion suggested a potential for developing these peptides as antagonists of PA infection, which should be addressed in future studies as well.

Lastly, the significance of ectoATP5B in VECs goes beyond bacterial infection. As mentioned earlier, HIV-1 or HEV might also bind ectopic ATP synthase in some ways (Liang et al., 2010; Yavlovich et al., 2012; Ahmed et al., 2016). In a recent review article about the COVID-19 pandemic, Panfoli proposed that the endothelial surface ectopic redox complexes, including the ectopic ATP synthase, might be involved in the pathogenesis of critical COVID-19 (Panfoli, 2020). Currently, it is well taken proven SARS-CoV-2 viremia is a decisive factor for COVID-19 to progress to the critical stage or mortality (Bermejo-Martin et al., 2020; Hagman et al., 2020; Veyer et al., 2020), but many links are missing between the SARS-CoV-2 virus and those life-threatening vascular damages. To us, in light of the responsive features of ectopic ATP synthase on VECs to pathogens (e.g., aforementioned viruses) or pathogen-derived molecules (e.g., FimH and FlgE), the possibility for SARS-CoV-2 to utilize ectopic ATP synthase on VECs as a receptor could not be excluded.

In summary, the current findings that PAO1 FlgE targeted ectoATP5B to modulate VECs and vascular properties shed new lights on how PA or pathogens interact with a host. These data suggested FlgE as a player during bacteria–host or flagellum–VEC interactions *via* ectoATP5B and might be revealing a new pathway underlying the pathogenesis of infection-related diseases. Should the hypothetical contribution of FlgE–ectoATP5B axis to any VEC-associated disorders be confirmed, novel strategy or therapeutics targeting these counterparts might be developed for a better management of such health problems.

## Data Availability Statement

The raw data supporting the conclusions of this article will be made available by the authors, without undue reservation.

## Ethics Statement

The animal study was reviewed and approved by the ethics committee of The First Affiliated Hospital of Soochow University.

## Author Contributions

YW conceived the study. YW, YL, and SJ designed the experiments. YL, YS, YZ, MW, and QH performed the experiments. YL, QH, and YT supplied part of the reagents. BW performed the 3D structural analysis. YL and SJ did the statistical analysis. YL, YS, and YW prepared the manuscript. All authors contributed to the article and approved the submitted version.

## Funding

This work was supported by a grant from the National Natural Science Foundation of China (grant number 81571544). YW was partially supported by a starting package of Xiang’an Hospital of Xiamen University (PM20205180001).

## Conflict of Interest

The authors declare that the research was conducted in the absence of any commercial or financial relationships that could be construed as a potential conflict of interest.

## Publisher’s Note

All claims expressed in this article are solely those of the authors and do not necessarily represent those of their affiliated organizations, or those of the publisher, the editors and the reviewers. Any product that may be evaluated in this article, or claim that may be made by its manufacturer, is not guaranteed or endorsed by the publisher.
