# Supplementary material for: Flagellar Hook Protein FlgE Induces Microvascular Hyperpermeability via Ectopic ATP Synthase β on Endothelial Surface
Source: Front Cell Infect Microbiol. 2021 Nov 2;11:724912. doi: 10.3389/fcimb.2021.724912 (PMC8593108; doi:10.3389/fcimb.2021.724912)
Supplement: Supplementary file 1 [file DataSheet_1.docx]

Supplementary Material/Methods

# Cell surface ATP generation assay

Cell surface ATPase-driven ATP production was evaluated using a luminescence assay (Arakaki et al., 2003). In brief, HUVECs and SVECs grown in 24-well plate were washed with HEPES buffer (10 mM HEPES pH 7.4, 150 mM NaCl). Cells were treated with 0.2 mL HEPES buffer containing 2 mM MgCl2 with or without anti-ATP5B antibodies (50 μg/mL), FlgE (5 μg/mL, 10 μg/mL, 20 μg/mL), FlgEM (5 μg/mL, 10 μg/mL, 20 μg/mL), Pc-B (0.1 μM, 1μM, 10 μM), or Pc-F (0.1 μM, 1 μM, 10 μM) at 37°C for 1 h, followed by addition of 100 μM ADP (Sigma) supplemented with 10 mM potassium phosphate. Fifteen seconds later, the supernatant was harvested and ATP contents in it were measured using an Enhanced ATP Assay Kit according to the manufacturer’s instruction in a BioTeK Synergy H1 plate reader.

# Proliferation assay of VECs after FlgE/FlgEM treatment

HUVECs and SVECs (10^7^ cells/mL) were labeled with 5 μM CFDA SE (Carboxyfluorescein diacetate, succinimidyl ester, Beyotime, Shanghai, China) seeded in 6-well plates (5×10^4^ cells/well) and pretreated with anti- ATP5B antibodies at 50 μg/mL for 1 h. Then FlgE or FlgEM proteins were added to 20 μg/mL, or peptides Pc-B or Pc-F to 100 μM, and incubation continued for 48 h. The cells were harvested for a Beckman flow cytometer (CyAn ADP 9C, Beckman) at 488 nm and the data of proliferation were calculated using FlowJo 10 (Tree Star, Ashland, OR). By essence of this methodology, mean fluorescence intensity (MFI) was used directly to reflect the proliferation activity of the cell polulation, with lower MFI indicating more proliferation went through.

# Apoptosis assay of VECs after FlgE/FlgEM treatment

HUVECs and SVECs were cultured in 6-well plates (5×10^4^ cells/well) till confluence and starved for 4 h in culture medium without bovine serum, and then incubated for 1 h with 50 μg/mL anti-ATP5B antibodies or not, followed by 20 μg/mL FlgE or FlgEM stimulation for another 4 h. Then cells were harvested and stained with PE-Annexin V Apoptosis Detection Kit I following manufacturer’s instruction. Flow cytometry was run and data analyzed as described above.

# Lactate dehydrogenase release measurements of VECs after FlgE/FlgEM treatment

HUVECs and SVECs were seeded in 96-well plates (10^4^ cells/100 μL) for 12 h and then switched to serum-free medium. Pretreatment with anti-ATP5B antibodies at 50 μg/mL lasted for 1 h. Then FlgE or FlgEM proteins were added to 20 μg/mL and incubation continued for 12 h. After spinning, supernatants (60 μL/well) were transferred to a new plate and lactate dehydrogenase were measured using a LDH Cytotoxicity Assay Kit. Negative and positive controls were set as recommended by the kit manufacture.

# Cell migration assay

Migration assay was performed in 24-well plates using a scratch model. In brief, HUVECs were grown to full confluence and switched to serum-free medium and incubated for 1 h with 50 μg/mL anti-ATP5B antibodies or not. A scratch was made using a yellow tip across the monolayer and deattached cells were removed by washing. The remaining cells were cultured with 20 μg/mL FlgE or FlgEM for 8 h. The images were captured under microscope at same places of the scratch before and after culture, and the migrated areas were analyzed using Image J software.

# Endothelial tube formation assay

Tube-like structure formation of VECs was measured as described before (Okazaki et al., 2011). In brief, 50 μL Matrigel was added in 96-well plate and allowed to solidify at 37°C for 30 min. HUVECs were treated with or without various reagents (i.e. anti-ATP5B antibodies 50 μg/mL, FlgE or FlgEM 10 μg/mL, or combination of anti-ATP5B with FlgE or FlgEM) and seeded at a density of 2×10^4^ cells/well. Four hours later, images were captured using a microscope. Capillary-like tubes were traced and the total tube length of each group was calculated by Image J software.

# Generation, purification and characterization of polyclonal rabbit anti-FlgE antibodies.

Design and production of recombinant FlgE proteins were described earlier in detail (Shen et al., 2017). For production of anti-FlgE antibodies, New Zealand White rabbits (female, 2.8 kg of body weight) were immunized with 2 mg FlgE proteins in Complete Freund's Adjuvant at hind footpads as the first dose, followed by injection of 2 mg FlgE proteins in Incomplete Freund's Adjuvant into palpable popliteal lymphnodes as the second dose at day 7, and another 2 mg FlgE proteins in PBS adjuvant via intravenous injection at day 21 as the third dose. Three more weeks later, the rabbits were sacrificed and serum were collected. The titers of anti-FlgE Ab in serum were measure with ELISA on purified recombinant FlgE proteins (5 μg/mL)-coated plates. IgG antibodies were purified via saturate ammonia sulphate precipitation and dialysis against PBS following routine procedure. The specificity of the antibodies was confirmed using recombinant FlgE and PAO1 lysate (Supplementary Figure S1)

.

***Supplementary Figures***


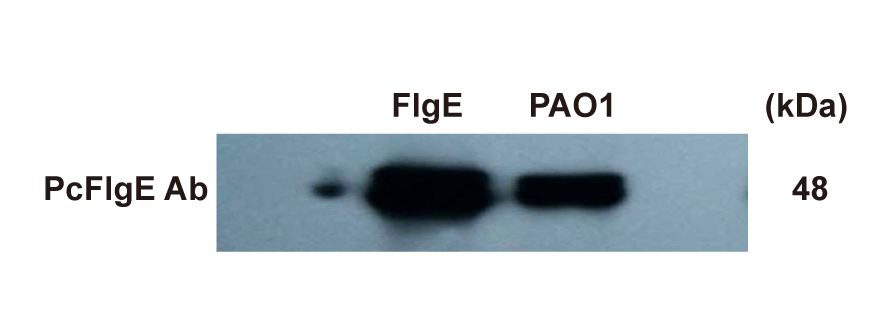


**Figure S1. Immunoblotting of recombinant FlgE and PAO1 lysate with rabbit polyclonal anti-FlgE antibodies to confirm the specificity of polyclonal rabbit anti-FlgE antibodies.** In brief, PAO1 were grown in LB medium at 37ºC to an optical density of 0.5 at OD600 before harvest. Whole-cell lysate and purified recombinant FlgE protein (50 μg) were resolved in SDS-PAGE gels and subjected to immunobloting with PcFlgE Ab (1 μg/mL). Blotting results demonstrated that the rabbit polyclonal anti-FlgE detected FlgE exclusively at the desired position.

***Table S1 Primer pairs for gene expression used in the study.***

| **Gene (human)** | **Primers** | **Sequence** |
| --- | --- | --- |
| Il-1β | Forward | 5’-CAACCAACAAGTGATATTCTCCATG-3’ |
|  | Reverse | 5’-CAACCAACAAGTGATATTCTCCATG-3’ |
| IL-6 | Forward | 5’-TCGGAGGCTTAATTACACATGTTC-3’ |
|  | Reverse | 5’-CAAGTGCATCATCGTTGTTCATAC-3’ |
| Mmp2 | Forward | 5’-GGTTGTCTGAAGTCACTGCACAGT-3’ |
|  | Reverse | 5’-CTCGGTAGGGACATGCTAAGTAGAG-3’ |
| Mmp9 | Forward | 5’-CCTGGAGACCTGAGAACCAATCT-3’ |
|  | Reverse | 5’-CCACCCGAGTGTAACCATAGC-3’ |
| Timp-1 | Forward | 5’-CTTCTGCAATTCCGACCTCGT-3’ |
|  | Reverse | 5’-ACGCTGGTATAAGGTGGTCTG-3’ |
| Timp-2 | Forward | 5’-GGATGGACTGGGTCACAGCGCA-3’ |
|  | Reverse | 5’-TTCTCTTGATGCAGGCGCCGC-3’ |
| β-Actin | Forward | 5’-TGGCACCCAGCACACTGAA-3’ |
|  | Reverse | 5’-CTAAGTCATAGTCCGCCTAGAAGCA-3’ |

# *Table S2 Calculated interaction forces between FlgE and ATP5B.*

| **Energy** | **FlgE/ATB5B** | **SARS-CoV-2/ACE2** | **SARS-CoV/ACE2** |
| --- | --- | --- | --- |
| Electrostatic interaction (kcal/mol) | -672 | -732 | -719 |
| Van der Waals interaction (kcal/mol) | -158 | -96 | -95 |
| Total | -830 | -828 | -814 |
| Reference | This study | [a] | [a] |

1. [Spinello](https://www.ncbi.nlm.nih.gov/pubmed/?term=Spinello%20A%5BAuthor%5D&cauthor=true&cauthor_uid=32463239) A, [Saltalamacchia](https://www.ncbi.nlm.nih.gov/pubmed/?term=Saltalamacchia%20A%5BAuthor%5D&cauthor=true&cauthor_uid=32463239) A, [Magistrato](https://www.ncbi.nlm.nih.gov/pubmed/?term=Magistrato%20A%5BAuthor%5D&cauthor=true&cauthor_uid=32463239) A. Is the Rigidity of SARS-CoV-2 Spike Receptor-Binding Motif the Hallmark for Its Enhanced Infectivity? Insights from All-Atom Simulations. [J Phys Chem Lett](https://www.ncbi.nlm.nih.gov/pmc/articles/PMC7274147/). 2020; 11: 4785–4790.

**References**

Arakaki, N., Nagao, T., Niki, R., Toyofuku, A., Tanaka, H., Kuramoto, Y., et al. (2003). Possible role of cell surface H+ -ATP synthase in the extracellular ATP synthesis and proliferation of human umbilical vein endothelial cells. *Mol Cancer Res* 1(13)**,** 931-939.

Okazaki, H., Tokumaru, S., Hanakawa, Y., Shiraishi, K., Shirakata, Y., Dai, X., et al. (2011). Nuclear translocation of phosphorylated STAT3 regulates VEGF-A-induced lymphatic endothelial cell migration and tube formation. *Biochem Biophys Res Commun* 412(3)**,** 441-445. doi: 10.1016/j.bbrc.2011.07.111.

Shen, Y., Chen, L., Wang, M., Lin, D., Liang, Z., Song, P., et al. (2017). Flagellar Hooks and Hook Protein FlgE Participate in Host Microbe Interactions at Immunological Level. *Sci Rep* 7(1)**,** 1433. doi: 10.1038/s41598-017-01619-1.
